# Safety and Duration of Effect of 40-Unit PrabotulinumtoxinA-xvfs for the Treatment of Moderate to Severe Glabellar Lines in Adult Patients: A Phase II, Multicenter, Randomized, Double-Blind, Active-Controlled Trial

**DOI:** 10.1093/asj/sjae051

**Published:** 2024-03-20

**Authors:** Steven Fagien, Rui L Avelar, Sue Ellen Cox, John H Joseph, Joely Kaufman-Janette, Keith A Marcus

## Abstract

**Background:**

Extending the duration of effect of botulinum toxins—by administering doses beyond those of the approved labels—has been an area of increasing interest in the field of aesthetics.

**Objectives:**

The aim of this study was to investigate the safety and duration of effect of 40-unit (U) prabotulinumtoxinA-xvfs (twice the approved dose and concentration) for the treatment of moderate-to-severe glabellar lines.

**Methods:**

A total of 154 adult patients were randomized 1:1:1 to a single treatment of either 40 U prabotulinumtoxinA-xvfs (PRA 40, 5 injections of 8 U/0.05 mL), or 20 U of either prabotulinumtoxinA-xvfs (PRA 20) or onabotulinumtoxinA (ONA 20). Both 20-U controls were administered as 5 injections of 4 U/0.1 mL. Efficacy and safety were assessed on days 2, 7 (by telephone), 30, and every 30 days thereafter up to 365 days or until the patient had returned to baseline. The primary effectiveness endpoint was the duration of effect (estimated by Kaplan-Meier analysis), defined as the number of days from treatment day (baseline) to the day that glabellar line severity at maximum frown by investigator assessment returned to the baseline value.

**Results:**

Patients had a mean age of 47 years (20-72 years); 69.5% had severe glabellar lines at baseline. Of the 36 adverse events, 32 (88.9%) were mild and 4 (11.1%) were moderate in severity; none were serious. The median durations of effect were estimated to be 183, 149, and 148 days for PRA 40–, PRA 20–, and ONA 20–treated patients, respectively.

**Conclusions:**

In this phase 2 pilot study, 40 U prabotulinumtoxinA-xvfs was observed to be safe and had a duration of 6 months.

**Level of Evidence: 1:**

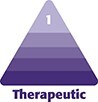

Since first introduced over 20 years ago, botulinum toxin/neuromodulator injections have become the top minimally invasive cosmetic procedure performed in the United States and elsewhere. As reported by The Aesthetic Society, based on the National Databank statistics, a total of 3,945,282 toxin procedures were performed in 2022, representing an increase of 24% from 2021.^1^

Currently, a total of 5 botulinum toxin type A formulations are approved in the United States for the temporary improvement in the appearance of moderate to severe glabellar lines associated with corrugator and/or procerus muscle activity in adult patients. These include: 20-unit (U) onabotulinumtoxinA (Botox Cosmetic; Allergan Inc., Ireland; initial approval 1989); 50 U abobotulinumtoxinA (Dysport; Ipsen Biopharm Ltd., Wrexham, UK; approved 2009); 20 U incobotulinumtoxinA (Xeomin; Merz Pharmaceuticals GmbH, Frankfurt, Germany; approved 2011); 20 U prabotulinumtoxinA-xvfs (Jeuveau; Evolus, Inc., Newport Beach, CA; approved 2019); and 40 U daxibotulinumtoxinA-lanm (Daxxify; Revance Therapeutics, Inc., Newark, CA; approved 2022).^2-6^ At approved doses, the cosmetic benefits of these neuromodulators are evident within days of injection, with peak effects typically reached within 30 days; when stated, on-label FDA duration of effect claims range from 3 to 4 months.^2-6^

Given the popularity of these products and the fact that many patients are long-term repeat customers, it is not surprising that there has been a considerable interest in investigating how a prolonged effect and less frequent, more consumer-friendly treatment schedule might be achieved. The focus of these investigations has been to study higher and more concentrated doses of toxins as a means of increasing their duration of effect. In fact, clinical studies reporting on doses up to 5-fold that of the approved dose for the treatment of glabellar lines have been published in recent years with varying results.^7-15^ Based on reviews of these data, it has generally been concluded that prolonged durations of effect are indeed achieved with higher doses of toxin and that individualized treatment plans, often at higher than approved doses, may well be the optimal approach in future patient care.^16,17^ At the same time, it is clear from these multidose studies that the relationship between dose and duration is nonlinear and that a plateau effect develops after which none or minimal benefit is achieved with higher doses of toxin.

The current study (EV-201) was the first to report on investigation of a higher dose, hyperconcentrated formulation of prabotulinumtoxinA-xvfs. Based in part on the dose response observed with onabotulinumtoxinA and daxibotulinumtoxinA-lanm (see Discussion), and the fact that both onabotulinumtoxinA and prabotulinumtoxinA-xvfs are 900 kDa molecules, a dose of 40 U was selected for this initial investigation.^9,14^ This was a phase 2, active-controlled pilot study undertaken with the objective of demonstrating the safety and duration of effect of 40 U prabotulinumtoxinA-xvfs—that is, at twice the approved dose and concentration—in providing temporary improvement in the appearance of moderate to severe glabellar lines in adult patients.

## METHODS

### Study Design and Conduct

EV-201 was a prospectively designed, multicenter, randomized, double-blind, active-controlled, single-treatment study of up to 379 days duration; onabotulinumtoxinA and prabotulinumtoxinA-xvfs, each at the approved dose of 20 U, served as the active controls. The EV-201 study was conducted at 5 investigative sites in the United States between March 2022 and May 2023. The study protocol and its subsequent revision were approved by a single centralized institution review board, Veritas IRB, Kirkland, QC, Canada. The study was conducted in compliance with applicable sections of the US Title 21 Code of Federal Regulations, commonly known as Good Clinical Practices, and consistent with the ethical principles that have their origin in the Declaration of Helsinki. ClinicalTrials.gov identifier: NCT05320393.

### Patients

Study patients were selected from a population of healthy adults, at least 18 years of age, who had moderate to severe glabellar lines at maximum frown as assessed by both the investigator and the participant with the validated 4-point photonumeric Glabellar Line Scale (GLS; 0 = none, 1 = mild, 2 = moderate, 3 = severe). All provided written informed consent before entering the study. Prospective participants were excluded for a variety of reasons including: females who were pregnant, breastfeeding, or of childbearing potential and not practicing reliable birth control; and individuals who had any medical or psychiatric condition, previous treatment, or history that might have interfered with study participation or the interpretation of results. Examples of these latter exclusion criteria included: current, or history of, eyelid or eyebrow ptosis; previous treatment with a botulinum toxin of any serotype above the level of the lateral canthus within the previous 6 months; and, inability to substantially lessen glabellar frown lines even by physically spreading them apart.

### Treatments and Follow-Up

On treatment day, GLS severity at maximum frown was assessed by the investigator and patient to confirm eligibility and establish baseline GLS values. Patients were then randomly assigned in a 1:1:1 ratio to receive a single treatment of either 40 U prabotulinumtoxinA-xvfs, 20 U prabotulinumtoxinA-xvfs, or 20 U onabotulinumtoxinA. Random numbers were generated utilizing R Software; a block randomization scheme was employed, with blocks stratified by baseline GLS score at maximum frown (moderate vs severe), age (≤65 vs >65 years) and sex (female vs male). Unblinded site personnel (eg, study nurse) accessed the randomization schedule and reconstituted the assigned study treatment to a final dilution of: 4 U/0.1 mL for those patients randomized to 1 of the active controls; or, 8 U/0.05 mL for those patients randomized to 40 U prabotulinumtoxinA-xvfs. An unblinded injector, who was not responsible for patient follow-up, administered the assigned study treatment in accordance with the approved prescribing information for these toxins by intramuscular injection (at a depth based on their expertise) into each of 5 target injection sites: the midline of the procerus, the inferomedial aspect of each corrugator muscle, and the superior middle aspect of each corrugator, at least 1 cm above the bony orbital rim. The 20-U active controls were injected as 0.1 mL (4 U) per target site; 40 U prabotulinumtoxinA-xvfs was injected as 0.05 mL (8 U) per target site.

Safety and effectiveness outcomes were subsequently assessed on posttreatment days 2 (48 hours), 7 (by telephone) and 30, and then every 30 days up to 365 days or until the participant had returned to their pretreatment baseline GLS value as assessed by the investigator, at which point the participant exited the study. With the intent of improving patient compliance over the yearlong study, no clinic visits were scheduled in the early posttreatment phase between days 2 and 30. To ensure blinding throughout this study, the investigator, who was responsible for managing the patients, monitoring for related and unrelated adverse events, and performing all safety and effectiveness evaluations, remained blinded to the randomized treatment throughout the study (as did the patient).

### Outcomes and Statistical Analysis

Key efficacy measures included: glabellar lines at maximum frown as measured by investigator and patient on the GLS; aesthetics as measured by investigator and patient on the 5-point Global Aesthetic Improvement Scale (GAIS, 2 = much improved, 1 = improved, 0 = no change, −1 = worse, −2 = much worse); and, satisfaction as measured by the patient on the 5-point Subject Satisfaction Scale (SSS, 2 = very satisfied, 1 = satisfied, 0 = indifferent, −1 = unsatisfied, −2 = very unsatisfied). Key safety measures included investigator assessment of adverse events.

The primary and select other effectiveness endpoints were measures of the duration of effect, described by Kaplan-Meier curves and the respective median times, with associated 2-sided 95% confidence intervals (CIs) for each treatment group. The primary effectiveness endpoint was the duration of effect (evaluated for all patients), defined as the number of days from treatment day (baseline) to the day that GLS severity at maximum frown returned to the baseline value, by investigator assessment; this measure by patient assessment was a secondary endpoint. The study treatment was considered to have remained effective for the time during which GLS severity at maximum frown had not returned to the baseline value. Duration of effect was further evaluated, by each investigator and patient assessment, for subsets of patients who met various responder definitions, including: (1) patients who achieved a ≥ 1-point improvement on the GLS at maximum frown at any point during the study; and, (2) patients who were deemed GAIS responders, defined as patients who achieved a GAIS score of 1 or 2 at Day 30. Effectiveness was also examined as the percentage of responders over time, again based on subsets of patients who met various responder definitions.

The primary population for analysis of effectiveness outcomes was the modified intent-to-treat population (mITT), defined as all patients who were randomized, received their injection of investigational product, and had at least 1 postdosing assessment for the primary effectiveness endpoint. For duration of effect measures, statistical analyses of treatment group differences were done with Cox proportional hazards regression with time to return to baseline as the dependent variable, treatment group as the independent variable, and age group and sex as covariates. For those analyses in which duration of effect was based on the GLS, the respective baseline GLS at maximum frown by either investigator or patient assessment also served as covariates. Statistical significance was established at the 0.025 level with 1-sided hypothesis testing. For responder rate over time measures, odds ratios and *P* values for differences in responder rates at each study visit were calculated by Fisher's exact test. Note that this study was not powered for formal hypothesis testing; analyses were intended for interpretation purposes only.

All treatment-related adverse events, defined by the investigator as possibly, probably, or definitely treatment related, were summarized. These included all events, any serious events, and most common events (ie, headache). The primary population for presentation of safety outcomes was the safety population, defined as all patients who were randomized and received treatment with investigational product. No statistical analyses of safety data were performed.

### Sample Size

This study was not powered for inferential analyses. Assuming a total of 150 patients, with 50 patients randomized per treatment group, exponential distributions for failure and censuring times, a median duration of effect of 4 months in the control groups, and an annual censoring rate of 10%, the study was estimated to have 80% power to detect a hazard ratio of 0.47 based on a 1-sided hypothesis test of the primary effectiveness endpoint with alpha = 0.025. Therefore it was expected that this sample size would be sufficient to address the study objectives.

## RESULTS

### Patient Disposition and Demographics

**Figure 1.**
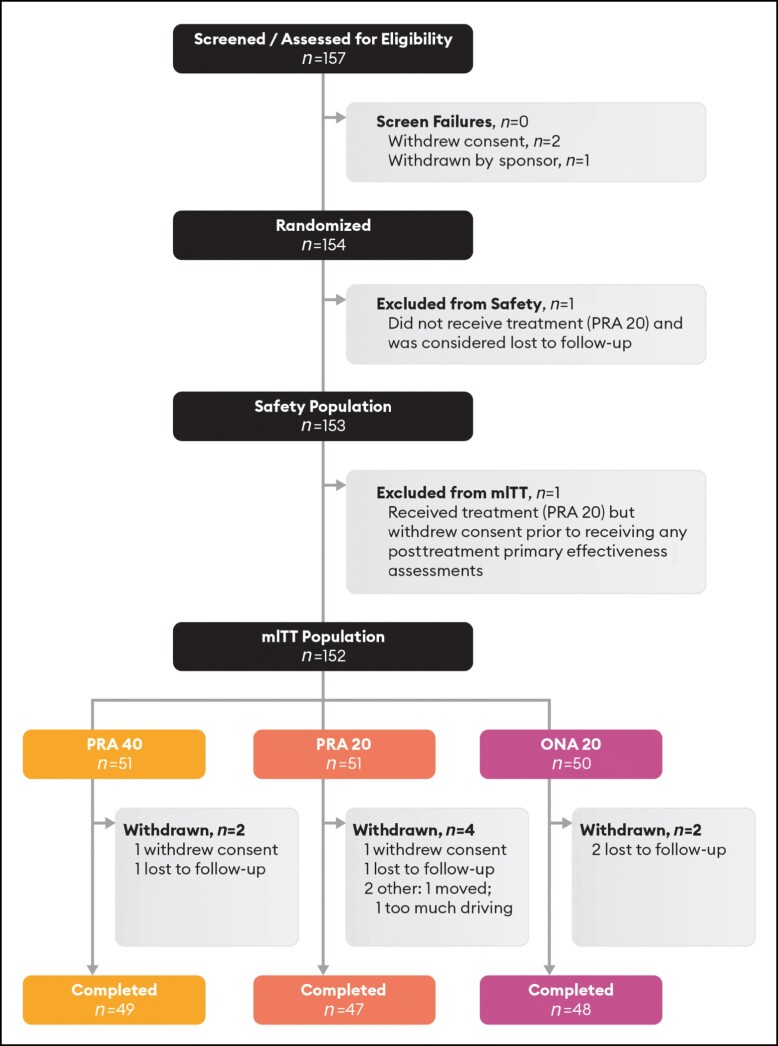
CONSORT flow diagram. Randomized: all participants who were randomized to treatment; safety population: all randomized participants who received treatment with investigational product; mITT population: all participants who were randomized, received their injection of investigational product, and had at least 1 postdosing assessment for the primary effectiveness endpoint. ONA, onabotulinumtoxinA; PRA, prabotulinumtoxinA-xvfs.

A total of 154 patients were randomized to treatment (Figure 1); of these, 1 patient did not receive treatment and was excluded from the safety population (*n* = 153), and a second patient withdrew before receiving any posttreatment assessment and was excluded from the mITT population (*n* = 152). Of the 154 randomized, 51 (33.1%) received treatment with 40 U prabotulinumtoxinA-xvfs (PRA 40); 53 (34.4%) with 20 U prabotulinumtoxinA-xvfs (PRA 20); and 50 (32.5%) with 20 U onabotulinumtoxinA (ONA 20). In total, 144 patients (93.5% of all randomized; 94.7% of all mITT participants) attended their final visit and completed the study at the time that their GLS score at maximum frown by investigator assessment returned to their pretreatment baseline GLS value. Ten participants (2/51 randomized to PRA 40, 3.9%; 6/53 randomized to PRA 20, 11.3%; 2/50 randomized to ONA 20, 4.0%) were withdrawn. Most commonly (8/10), these patients were lost to follow-up or withdrew consent. No patient was withdrawn due to an adverse event. Posttreatment, PRA 40 patients were followed for a mean of 189 days (range 56-333), whereas PRA 20 and ONA 20 patients were followed for means of 155 days (range of 37-272) and 152 days (range of 79-280), respectively. The last PRA 40 patient exited the study at the Day 330 visit, and both the last PRA 20 and ONA 20 patients exited the study at their Day 270 visits.

PRA 40, PRA 20, and ONA 20 groups were similar in their demographic characteristics at baseline (Table 1). Randomized participants had a mean age of approximately 47 years (range 20 to 72 years). Most (94.2%) were female; most (93.5%) were racially identified as White. Approximately one-third (33.1%) were of Hispanic or Latino ethnicity. Many had a previous history of exposure to botulinum toxins. More participants had severe, rather than moderate, glabellar lines at maximum frown at baseline: 69.5% had a GLS score of 3 (ie, severe) by investigator assessment; 79.9% did by participant assessment.

**Table 1.** Baseline Demographic and Glabellar Line Characteristics—All Randomized (*n* = 154)

Characteristic	PRA 40 (*n* = 51)	PRA 20 (*n* = 53)	ONA 20 (*n* = 50)
Age (years)			
mean ± SD	46.8 ± 11.3	46.8 ± 12.9	47.0 ± 11.1
(min, max)	(20, 69)	(22, 72)	(25, 72)
Sex, *n* (%)			
Male	3 (5.9)	3 (5.7)	3 (6.0)
Female	48 (94.1)	50 (94.3)	47 (94.0)
Race, *n* (%)			
White	48 (94.1)	49 (92.5)	47 (94.0)
Black or African American	3 (5.9)	3 (5.7)	2 (4.0)
Asian	0 (0.0)	1 (1.9)	1 (2.0)
Other	0 (0.0)	0 (0.0)	0 (0.0)
Ethnicity, *n* (%)			
Hispanic or Latino	20 (39.2)	15 (28.3)	16 (32.0)
Not Hispanic or Latino	31 (60.8)	37 (69.8)	34 (68.0)
Not reported	0 (0.0)	1 (1.9)	0 (0.0)
History of toxin exposure, *n* (%)	26 (51.0)	19 (35.8)	26 (52.0)
GLS score at maximum frown			
Investigator assessment, *n* (%)			
Moderate	15 (29.4)	18 (34.0)	14 (28.0)
Severe	36 (70.6)	35 (66.0)	36 (72.0)
Patient assessment, *n* (%)			
Moderate	11 (21.6)	13 (24.5)	7 (14.0)
Severe	40 (78.4)	40 (75.5)	43 (86.0)

GLS, Glabellar Line Scale; max, maximum; min, minimum; ONA, onabotulinumtoxinA; PRA, prabotulinumtoxinA-xvfs; SD, standard deviation.

### Effectiveness

The median durations of effect of PRA 40, based on the primary and other effectiveness endpoints examined and estimated by Kaplan-Meier analyses, ranged between 183 and 184 days by investigator assessment, and between 178 and 183 by patient assessment (Tables 2, 3), that is, approximately 6 months, regardless of the endpoint examined and whether the assessor was the investigator or patient.

**Table 2.** Duration of Effect Outcomes Based on the GLS—mITT Population (*n* = 152)

No. days from treatment day to day GLS severity at maximum frown returned to baseline value	Median no. days^c^			Adjusted hazard ratio^d^	
	PRA 40 (*n* = 51)	PRA 20 (*n* = 51)	ONA 20 (*n* = 50)	PRA 40 vs PRA 20	PRA 40 vs ONA 20
All patients regardless of responder status					
Investigator assessment	183^e^	149^e^	148	1.892^e^	2.38
95% CI	(153, 211)	(130, 174)	(121, 174)	(1.230, 2.910)	(1.54, 3.67)
Stratified log-rank *P* value^a^				.0109^e^	.0002
Patient assessment	178	173	154	1.385	1.76
95% CI	(153, 212)	(146, 190)	(121, 177)	(0.844, 2.274)	(1.08, 2.87)
Stratified log-rank *P* value^a^				.3418	.0254
Responders who achieved a ≥ 1-point improvement on the GLS at maximum frown^b^					
Investigator assessment	183	149	148	1.892	2.38
95% CI	(153, 211)	(130, 174)	(121, 174)	(1.230, 2.910)	(1.54, 3.67)
Stratified log-rank *P* value^a^				.0109	.0002
Patient assessment	178	173	154	1.385	1.76
** **95% CI	(153, 212)	(146, 190)	(121, 177)	(0.844, 2.274)	(1.08, 2.87)
** **Stratified log-rank *P* value^a^				.3418	.0254

CI, confidence interval; GLS, Glabellar Line Scale; mITT, modified intent-to-treat; ONA, onabotulinumtoxinA; PRA, prabotulinumtoxinA-xvfs.^a^Stratified by treatment group. ^b^At any time point during the study. ^c^Estimated by Kaplan-Meier analyses. ^d^Estimated with Cox proportional hazards regression model, with time to return to baseline as dependent variable, treatment group as independent variable, and sex, age group, and respective baseline GLS score where appropriate as covariates. ^e^Primary effectiveness endpoint data.

**Table 3.** Duration of Effect Outcomes Based on the GAIS—mITT Population (*n* = 152)

	Median no. days^c^			Adjusted hazard ratio^d^	
	PRA 40 (*n* = 51)	PRA 20 (*n* = 51)	ONA 20 (*n* = 50)	PRA 40 vs PRA 20	PRA 40 vs ONA 20
No. days patients were GAIS responders (score of 1 or 2 at Day 30)^a^					
Investigator assessment	184	149	148	2.096	2.39
95% CI	(153, 212)	(129, 174)	(121, 174)	(1.348, 3.261)	(1.51, 3.77)
Stratified log-rank *P* value^b^				.0006	.0002
Patient assessment	183	156	146	1.620	2.47
** **95% CI	(153, 238)	(146, 181)	(120, 168)	(0.984, 2.668)	(1.49, 4.12)
** **Stratified log-rank *P* value^b^				.0639	.0002

CI, confidence interval; GLS, Glabellar Line Scale; mITT, modified intent-to-treat; ONA, onabotulinumtoxinA; PRA, prabotulinumtoxinA-xvfs.^a^Score of 1 or 2 = improved or much improved, respectively. ^b^Stratified by treatment group. ^c^Estimated by Kaplan-Meier analyses. ^d^Estimated with Cox proportional hazards regression model, with time to return to baseline as dependent variable, treatment group as independent variable, and sex and age group as covariates.

#### Duration of Effect Endpoints Based on the GLS

For all patients (ie, regardless of any responder definition), the median number of days from treatment day (baseline) to the day that GLS severity at maximum frown returned to the baseline value, by investigator assessment, for the PRA 40, PRA 20, and ONA 20 groups was 183, 149, and 148 days, respectively (Table 2). The difference in the durations of effect between PRA 40 and PRA 20 by investigator assessment, derived by comparing the 2 survival curves, was statistically significant: adjusted hazard ratio of 1.892; *P* value of .0109. Similarly, the difference in the durations of effect between PRA 40 and ONA 20 by investigator assessment was statistically significant: adjusted hazard ratio of 2.38; *P* value of .0002. Differences in the durations of effect by patient assessment were only statistically significant between PRA 40 and ONA 20 (Table 2).

**Figure 2.**
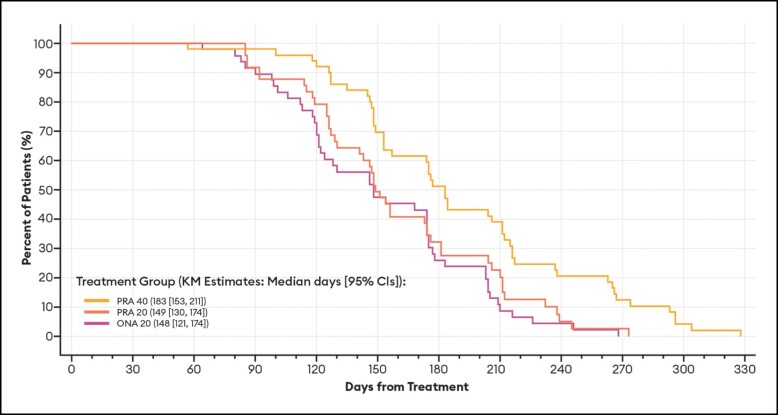
Kaplan-Meier plots of duration of effect for ≥1-point GLS responders, by investigator assessment—mITT population (*n* = 152). Data are presented for patients deemed responders at any point during the study by investigator assessment, in which a responder was defined as a patient who achieved at least a 1-point improvement on the GLS at maximum frown. Patients who had no effect were not included in the analysis. Duration of effect was calculated as the number of days from treatment day (baseline) to the day when the patient's GLS severity score at maximum frown returned to their baseline value. CI, confidence interval; GLS, Glabellar Line Scale; KM, Kaplan-Meier; mITT, modified intent-to-treat; ONA, onabotulinumtoxinA; PRA, prabotulinumtoxinA-xvfs.

**Figure 3.**
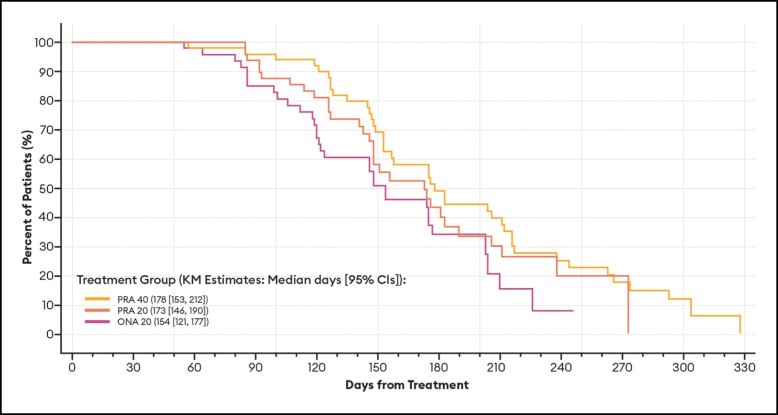
Kaplan-Meier plots of duration of effect for ≥1-point GLS responders, by patient assessment—mITT population (*n* = 152). Data are presented for patients deemed responders at any point during the study by patient assessment, in which a responder was defined as a patient who achieved at least a 1-point improvement on the GLS at maximum frown. Patients who had no effect were not included in the analysis. Duration of effect was calculated as the number of days from treatment day (baseline) to the day when the patient's GLS severity score at maximum frown returned to their baseline value. CI, confidence interval; GLS, Glabellar Line Scale; KM, Kaplan-Meier; mITT, modified intent-to-treat, ONA, onabotulinumtoxinA; PRA, prabotulinumtoxinA-xvfs.

Of particular note, the median duration of effect based on a return to baseline severity for the subset of patients who achieved a ≥ 1-point improvement on the GLS at maximum frown at any time during the study was identical to that estimated for all patients (Table 2). As is standard for these types of Kaplan-Meier calculations, patients who had no effect—in the current study, 1 PRA 20 and 1 ONA 20 patient by investigator assessment; 1 PRA 20 and 2 ONA 20 patients by patient assessment—were not included in the analyses. All others (150 by investigator assessment; 149 by patient assessment) were responders in that they achieved at least a 1-point improvement in their glabellar lines. Representative Kaplan-Meier plots for duration of effect based on a return to their baseline GLS severity score by investigator and patient assessments are presented in Figures 2 and 3, respectively.

#### Duration of Effect Endpoints Based on the GAIS

**Figure 4.**
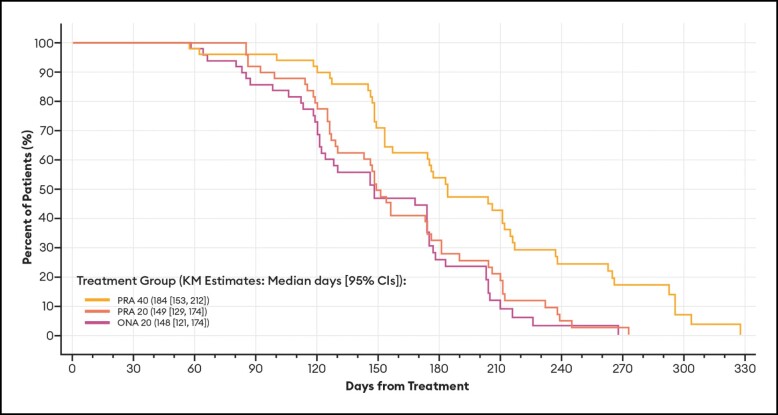
Kaplan-Meier plots of duration of effect for GAIS responders, by investigator assessment—mITT population (*n* = 152). Data are presented for patients deemed responders on or before Day 30 by investigator assessment, in which a responder was defined as a patient with GAIS score of 1 or 2 (1 = improved, 2 = much improved). Patients who had no effect were not included in the analysis. Duration of effect was calculated as the number of days from treatment day (baseline) to the day when the patient became a “nonresponder.” CI, confidence interval; GAIS, Global Aesthetic Improvement Scale; KM, Kaplan-Meier; mITT, modified intent-to-treat, ONA, onabotulinumtoxinA; PRA, prabotulinumtoxinA-xvfs.

**Figure 5.**
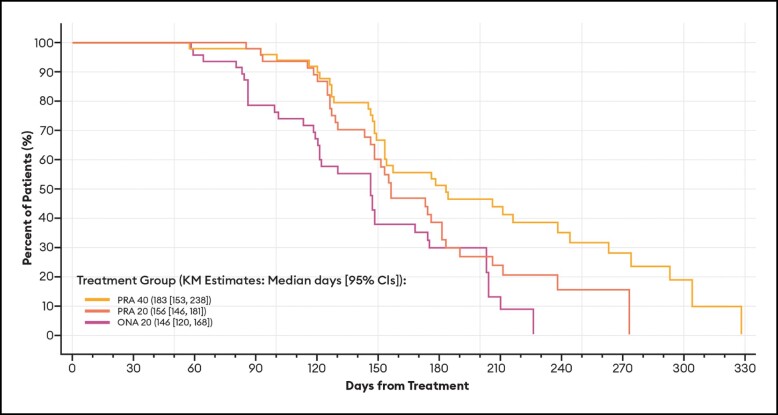
Kaplan-Meier plots of duration of effect for GAIS responders, by patient assessment—mITT population (*n* = 152). Data are presented for patients deemed responders on or before Day 30 by patient assessment, in which a responder was defined as a patient with GAIS score of 1 or 2 (1 = improved, 2 = much improved). Patients who had no effect were not included in the analysis. Duration of effect was calculated as the number of days from treatment day (baseline) to the day when the patient became a “nonresponder.” CI, confidence interval; GAIS, Global Aesthetic Improvement Scale; KM, Kaplan-Meier; mITT, modified intent-to-treat, ONA, onabotulinumtoxinA; PRA, prabotulinumtoxinA-xvfs.

As presented in the Table 3, and illustrated by the Kaplan-Meier plot (Figure 4), the difference in the durations of effect between PRA 40 and PRA 20 by investigator assessment was also statistically significant for the number of days participants were GAIS responders (score of 1 or 2 at Day 30): adjusted hazard ratio of 2.096; *P* value of 0.0006. As was observed for outcomes based on the GLS (Table 2), differences in the durations of effect between PRA 40 and ONA 20 were also statistically significant (*P*≤.05) for GAIS effectiveness endpoints by investigator assessment, while differences by patient assessment were only statistically significant between PRA 40 and ONA 20 (Table 3, Figures 4, 5).

#### Responder Rates Over Time Based on the GLS

**Figure 6.**
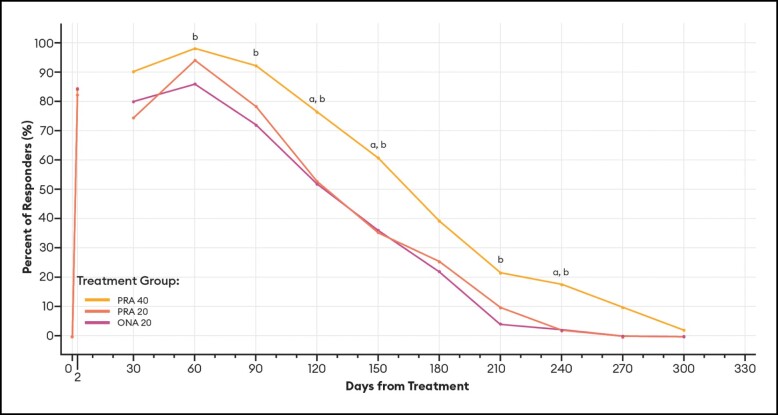
Responder rates for ≥1-Point GLS responders, by investigator assessment—mITT population (*n* = 152). Data are presented for patients deemed responders by investigator assessment, in which a responder was defined as a patient who achieved a ≥ 1-point improvement on the GLS at maximum frown. Note that no data were collected between Day 2 and Day 30. *P* values were calculated with Fisher's exact test. GLS, Glabellar Line Scale; mITT, modified intent-to-treat, ONA, onabotulinumtoxinA; PRA, prabotulinumtoxinA-xvfs. ^a^*P* < .05 PRA 40 vs PRA 20: Day 120 (76.5% vs 52.9%, *P* = .0220); 150 (60.8% vs 35.3%, *P* = .0170); and 240 (17.6% vs 2.0%, *P* = .0158). ^b^*P* < .05 PRA 40 vs ONA 20: Day 60 (98.0% vs 86.0%, *P* = .0310); 90 (92.2% vs 72.0%, *P* = .0097); 120 (76.5% vs 52.0%, *P* = .0129); 150 (60.8% vs 36.0%, *P* = .0170); 210 (21.6% vs 4.0%, *P* = .0148); and 240 (17.6% vs 2.0%, *P* = .0158).

**Figure 7.**
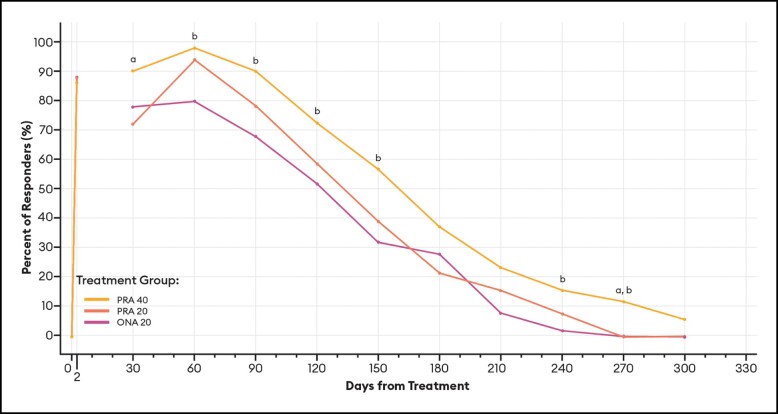
Responder rates for ≥1-Point GLS responders, by patient assessment—mITT population (*n* = 152). Data are presented for patients deemed responders by patient assessment, in which a responder was defined as a patient who achieved a ≥ 1-point improvement on the GLS at maximum frown. Note that no data were collected between Day 2 and Day 30. *P* values were calculated with Fisher's exact test. GLS, Glabellar Line Scale; mITT, modified intent-to-treat, ONA, onabotulinumtoxinA; PRA, prabotulinumtoxinA-xvfs. ^a^*P* < .05 PRA 40 vs PRA 20: Day 30 (90.2% vs 72.5%, *P* = .0400); and 270 (11.8% vs 0.0%, *P* = .0267). **^b^***P* < .05 PRA 40 vs ONA 20: Day 60 (98.0% vs 80.0%, *P* = .0038); 90 (90.2% vs 68.0%, *P* = .0072); 120 (72.5% vs 52.0%, *P* = .0410); 150 (56.9% vs 32.0%, *P* = .0163); 240 (15.7% vs 2.0%, *P* = .0310); and 270 (11.8% vs 0.0%, *P* = .0267).

For responders defined as patients with ≥ 1-point improvement in their GLS score at maximum frown, differences between PRA 40 and PRA 20 responder rates were statistically significant at Days 120, 150, and 240 by investigator assessment (Figure 6), and at Days 30 and 270 by patient assessment (Figure 7). Differences between PRA 40 and ONA 20 responder rates were statistically significant at each of Days 60, 90, 120, 150, 210, and 240 by investigator assessment, and at Days 60, 90, 120, 150, 240, and 270 by patient assessment (Figures 6, 7).

**Figure 8.**
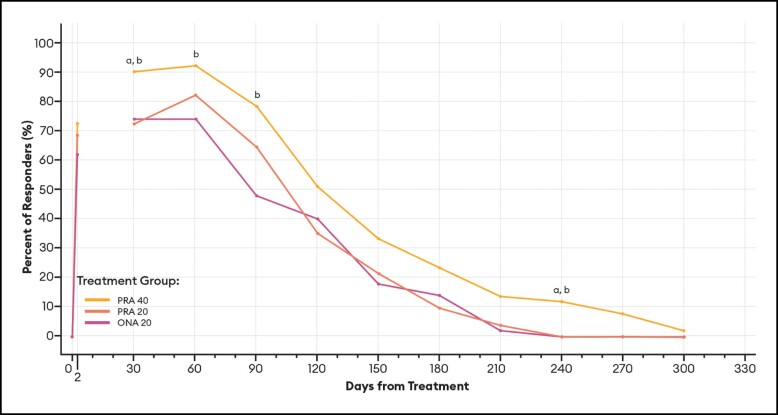
Responder rates for GLS score 0 or 1 responders, by investigator assessment—mITT population (*n* = 152). Data are presented for patients deemed responders by investigator assessment, in which a responder was defined as a patient with a GLS severity score of 0 or 1 (0 = none, 1 = mild) at maximum frown. Note that no data were collected between Day 2 and Day 30. *P* values were calculated with Fisher's exact test. GLS, Glabellar Line Scale; mITT, modified intent-to-treat, ONA, onabotulinumtoxinA; PRA, prabotulinumtoxinA-xvfs. ^a^*P* < .05 PRA 40 vs PRA 20: Day 30 (90.2% vs 72.5%, *P* = .0400); and 240 (11.8% vs 0.0%, *P* = .0267). ^b^*P* < .05 PRA 40 vs ONA 20: Day 30 (90.2% vs 74.0%, *P* = .0400); 60 (92.2% vs 74.0%, *P* = .0176); 90 (78.4% vs 48.0%, *P* = .0019); and 240 (11.8% vs 0.0%, *P* = .0267).

**Figure 9.**
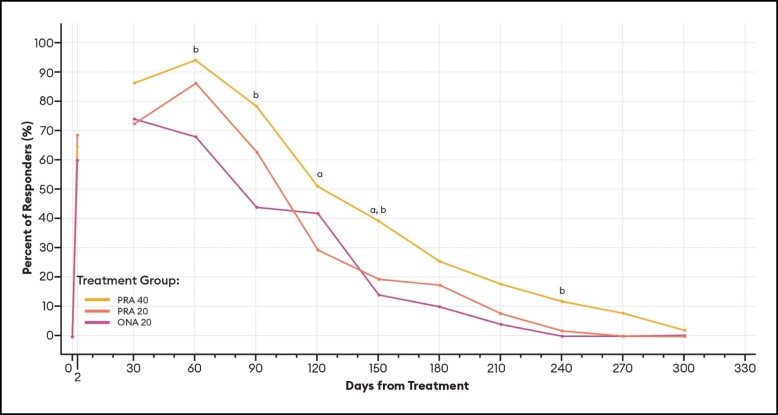
Responder rates for GLS score 0 or 1 responders, by patient assessment—mITT population (*n* = 152). Data are presented for patients deemed responders by patient assessment, in which a responder was defined as a patient with a GLS severity score of 0 or 1 (0 = none, 1 = mild) at maximum frown. Note that no data were collected between Day 2 and Day 30. *P* values were calculated with Fisher's exact test. GLS, Glabellar Line Scale; mITT, modified intent-to-treat, ONA, onabotulinumtoxinA; PRA, prabotulinumtoxinA-xvfs. ^a^*P* < .05 PRA 40 vs PRA 20: Day 120 (51.0% vs 29.4%, *P* = .0428); and 150 (39.2% vs 19.6%, *P* = .0495). ^b^*P* < .05 PRA 40 vs ONA 20: Day 60 (94.1% vs 68.0%, *P* = .0008); 90 (78.4% vs 44.0%, *P* = .0005); 150 (39.2% vs 14.0%, *P* = .0064); and 240 (11.8% vs 0.0%, *P* = .0267).

For responders defined as patients with a GLS score of 0 or 1 (none or mild) at maximum frown, differences between PRA 40 and PRA 20 responder rates were statistically significant at Days 30 and 240 by investigator assessment (Figure 8), and at Days 120 and 150 by patient assessment (Figure 9). Differences between PRA 40 and ONA 20 responder rates were statistically significant at Days 30, 60, 90, and 240 by investigator assessment, and at Days 60, 90, 150, and 240 by patient assessment (Figures 8, 9).

#### Responder Rates Over Time Based on the GAIS

**Figure 10.**
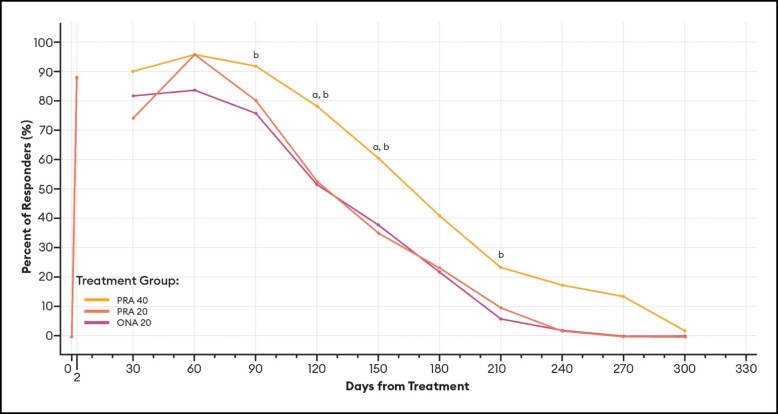
Responder rates for GAIS responders (score of 1 or 2), by investigator assessment—mITT population (*n* = 152). Data are presented for patients deemed responders by investigator assessment, in which a responder was defined as a patient with a GAIS score of 1 or 2 (1 = improved, 2 = much improved). Note that no data were collected between Day 2 and Day 30. *P* values were calculated with Fisher's exact test. GAIS, Global Aesthetic Improvement Scale; mITT, modified intent-to-treat, ONA, onabotulinumtoxinA; PRA, prabotulinumtoxinA-xvfs. ^a^*P* < .05 PRA 40 vs PRA 20: Day 120 (78.4% vs 52.9%, *P* = .0118); and 150 (60.8% vs 35.3%, *P* = .0170). ^b^*P* < .05 PRA 40 vs ONA: Day 90 (92.2% vs 76.0%, *P* = .0313); 120 (78.4% vs 52.0%, *P* = .0067); 150 (60.8% vs 38.0%, *P* = .0289); and 210 (23.5% vs 6.0%, *P* = .0228).

**Figure 11.**
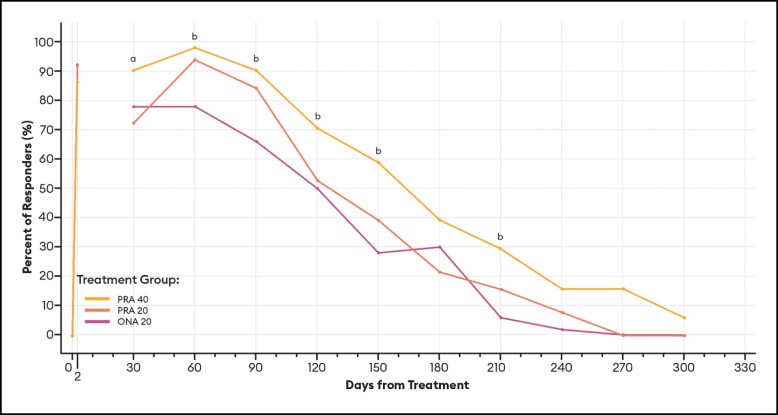
Responder rates for GAIS responders (score of 1 or 2), by patient assessment—mITT population (*n* = 152). Data are presented for patients deemed responders by patient assessment, in which a responder was defined as a patient with a GAIS score of 1 or 2 (1 = improved, 2 = much improved). Note that no data were collected between Day 2 and Day 30. *P* values were calculated with Fisher's exact test. GAIS, Global Aesthetic Improvement Scale; mITT, modified intent-to-treat, ONA, onabotulinumtoxinA; PRA, prabotulinumtoxinA-xvfs. ^a^*P* < .05 PRA 40 vs PRA 20: Day 30 (90.2% vs 72.5%, *P* = .0400). ^b^*P* < .05 PRA 40 vs ONA: Day 60 (98.0% vs 78.0%, *P* = .0018); 90 (90.2% vs 66.0%, *P* = .0038); 120 (70.6% vs 50.0%, *P* = .0428); 150 (58.8% vs 28.0%, *P* = .0025); and 210 (29.4% vs 6.0%, *P* = .0034).

For responders defined as patients who had a GAIS score of 1 or 2 (improved or very improved), differences between PRA 40 and PRA 20 responder rates were statistically significant at Days 120 and 150 by investigator assessment (Figure 10), and at Day 30 by patient assessment (Figure 11). Differences between PRA 40 and ONA 20 responder rates were statistically significant at each of Days 90, 120, 150, and 210 by investigator assessment, and at Days 60, 90, 120, 150, and 210 by patient assessment (Figures 10, 11).

#### Responder Rates Over Time Based on the SSS

**Figure 12.**
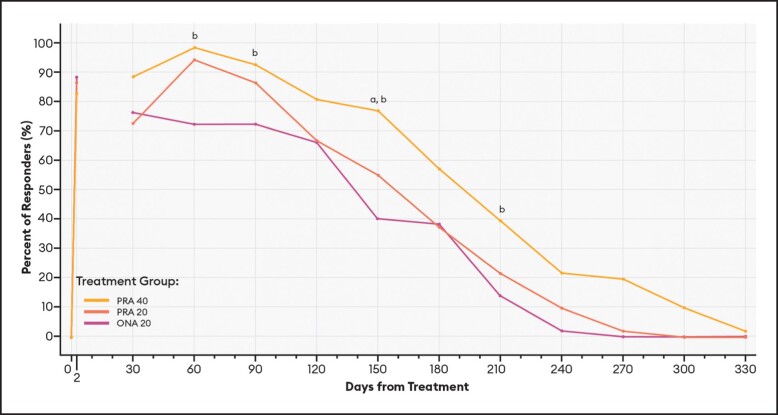
Responder rates for SSS responders (score of 1 or 2)—mITT population (*n* = 152). Data are presented for patients deemed responders, in which a responder was defined as a patient with an SSS score of 1 or 2 (1 = satisfied, 2 = very satisfied). Note that no data were collected between Day 2 and Day 30. *P* values were calculated with Fisher's exact test. mITT, modified intent-to-treat; ONA, onabotulinumtoxinA; PRA, prabotulinumtoxinA-xvfs; SSS, Subject Satisfaction Scale. ^a^*P* < .05 PRA 40 vs PRA 20: Day 150 (76.5% vs 54.9%, *P* = .0363). ^b^*P* < .05 PRA 40 vs ONA 20: Day 60 (98.0% vs 72.0%, *P* = .0002); 90 (92.2% vs 72.0%, *P* = .0097); 150 (76.5% vs 40.0%, *P* = .0003); and 210 (39.2% vs 14.0%, *P* = .0064).

For responders defined as patients with a SSS score of 1 or 2 (satisfied or very satisfied), differences between PRA 40 and PRA 20 responder rates were statistically significant at Day 150; differences between PRA 40 and ONA 20 responder rates were statistically significant at each of Days 60, 90, 150, and 210 (Figure 12).

### Safety

Safety data are reported for all 154 patients who received treatment (Table 4). No serious adverse events (including deaths) were reported. No unexpected adverse device effects were reported, and no participant experienced an adverse event that led to study discontinuation. Twenty-six participants (17.0%) experienced a total of 36 treatment-emergent adverse events over the course of this study. Within each group, 13.7% of PRA 40 participants, 15.4% of PRA 20 participants, and 22.0% of ONA 20 participants experienced 1 or more adverse events. By preferred term, the most common events, occurring at a frequency of 5% or more in any group, were headache and COVID-19. No participant experienced an event that was assessed as severe (data not displayed). Most events (32/36, 88.9%) were mild in severity. Of the 4 moderate events, 3 (COVID-19, urinary tract infection, cystitis) were not treatment related; see following for the single moderate treatment-related event. No pregnancies were reported.

**Table 4.** Summary of Adverse Events

Adverse event (AE) parameter by preferred term	PRA 40 (*n* = 51)		PRA 20 (*n* = 52)		ONA 20 (*n* = 50)	
	Events	*n* (%)	Events	*n* (%)	Events	*n* (%)
Any treatment-emergent AEs	8	7 (13.7)	16	8 (15.4)	12	11 (22.0)
Any serious AEs	0	0 (0.0)	0	0 (0.0)	0	0 (0.0)
Any unexpected adverse device effects	0	0 (0.0)	0	0 (0.0)	0	0 (0.0)
Any AE leading to study discontinuation	0	0 (0.0)	0	0 (0.0)	0	0 (0.0)
Most common AEs^a^						
Headache	2	2 (3.9)	4	3 (5.8)	2	2 (4.0)
COVID-19	0	0 (0.0)	0	0 (0.0)	3	3 (6.0)
Any treatment-related AEs	3	2 (3.9)	3	3 (5.8)	2	2 (4.0)
Eyelid ptosis	1	1 (2.0)	0	0 (0.0)	0	0 (0.0)
Facial discomfort	1	1 (2.0)	0	0 (0.0)	0	0 (0.0)
Headache	1	1 (2.0)	2	2 (3.8)	2	2 (4.0)
Presyncope	0	0 (0.0)	1	1 (1.9)	0	0 (0.0)

Adverse events, regardless of relationship or seriousness, were collected at all study visits and were reported in response to a query, observed by the investigator or site personnel, or reported spontaneously by the participant. ONA, onabotulinumtoxinA; PRA, prabotulinumtoxinA-xvfs. ^a^Frequency of ≥5% in any treatment group.

Seven participants (4.6%) experienced a total of 8 treatment-related adverse events (Table 4). The number of participants (either 2 or 3) who experienced a treatment-related event was similar across treatment groups: 2 (3.9%) in the PRA 40 group; 3 (5.8%) in PRA 20; and 2 (4.0%) in ONA 20. The only treatment-related adverse event experienced by more than 1 participant was headache: 5 of the 8 treatment-related events (62.5%) were headache (all mild in severity) and only 1 was reported for a participant in the PRA 40 group. A single case of eyelid ptosis (1/51, 2.0%) was observed in a 47-year-old White female in the PRA 40 group. This event, which was assessed as moderate in severity and treatment related, had an onset 15 days after treatment and a duration of 67 days; it resolved without sequelae. No particularly noteworthy differences were otherwise evident between the 3 treatment groups in the incidence of adverse events, or in the use of concomitant medications.

## DISCUSSION

This phase 2 dose-ranging study was undertaken to demonstrate the safety and duration of effect of 40 U prabotulinumtoxinA-xvfs in providing temporary improvement in the appearance of moderate to severe glabellar lines. No safety concerns related to the 40-U dose were evident at any point during this study. For all patients, the median duration of effect of 40 U prabotulinumtoxinA-xvfs, based on the return to baseline value of GLS severity at maximum frown, was consistently estimated to be 6 months (183 days—ie, 26.1 weeks) by investigator assessment. As has been reported previously with other toxins, the relationship between dose of prabotulinumtoxinA-xvfs and duration of effect was not a linear one.^16^ In the current study, based on the same GLS endpoint as above, the median durations of effect of 20 U prabotulinumtoxinA-xvfs and 20 U onabotulinumtoxinA were consistently estimated to be 149 and 148 days, respectively. That is, a 22.8% increase in the median duration of effect was achieved when the dose of prabotulinumtoxinA-xvfs was doubled from 20 to 40 U; a 23.6% increase in duration was achieved with the 40 U dose of prabotulinumtoxinA-xvfs compared with 20 U onabotulinumtoxinA. It may be that for select patients, an individualized treatment plan of 40 U prabotulinumtoxinA-xvfs at 6-month intervals will prove sufficient to maintain the desired degree of improvement in the appearance of their glabellar lines.

As evaluated with the Cox proportional hazards regression model (with sex, age, and baseline GLS score by investigator or patient assessment, where appropriate, as covariates), estimates of the duration of effect of 40 U prabotulinumtoxinA-xvfs, based on the primary and other effectiveness endpoints, were statistically significantly superior to those of the 20 U controls: superior to 20 U prabotulinumtoxinA-xvfs by investigator assessment only; superior to 20 U onabotulinumtoxinA by all investigator and patient assessments. Furthermore, as evident by comparing the adjusted hazards ratios, differences in the durations of effect between 40 U prabotulinumtoxinA-xvfs and 20 U onabotulinumtoxinA tended to be more pronounced than those between the 2 doses of prabotulinumtoxinA-xvfs, regardless of whether differences proved to be statistically significant or not. This trend was also evident in the various graphs illustrating responder rates over time, in which statistically significant differences in responder rates were more commonly observed by both investigator and patient assessment between 40 U prabotulinumtoxinA-xvfs and 20 U onabotulinumtoxinA.

Caution is always warranted when drawing any conclusions based on comparisons across studies rather than, as above, on direct within-study comparisons. It is important to be cognizant of differences in the severity and makeup of study populations and the potential impact of these differences on study outcomes. Inherent differences in study design, duration, doses selected, assessment scales, the choice of time points for those assessments, and sometimes subtle differences in responder definitions also limit our ability to interpret study results should we attempt to simply overlay graphed data.

With these caveats in mind, when examined for the subset of patients who achieved a ≥ 1-point improvement in their glabellar lines at maximum frown by investigator assessment, the median duration of effect reported for the 40-U dose of prabotulinumtoxinA-xvfs appears to compare favorably with durations reported with 40-U doses of other approved toxins for the treatment of glabellar lines. In the current study, as was observed for all patients, the median duration of effect of 40 U prabotulinumtoxinA-xvfs in this subpopulation was estimated to be 183 days (26.1 weeks) whereas a median duration of 149 days (21.3 weeks) was estimated for 20 U prabotulinumtoxinA-xvfs. That is, as noted, a 22.8% longer median duration of effect was observed when the dose was doubled to 40 U from the approved dose of 20 U. Importantly for the purposes of comparisons across studies, these durations of effect outcomes for the 20-U and 40-U doses were equally valid when other subsets were examined, including patients who achieved a glabellar line score of none or mild at maximum frown by investigator assessment. Of additional note, highly similar results were also observed when duration of effect was based on GAIS effectiveness endpoints.

Key 40-U dose comparator studies included those of onabotulinumtoxinA and daxibotulinumtoxinA-lanm.^9,14,15,18-20^ The first was a phase 2 dose-ranging study reporting on single doses of placebo and 20 U, 40 U, 60 U, and 80 U onabotulinumtoxinA.^9^ For those patients who achieved a ≥ 1-point improvement in their glabellar line severity at maximum frown by investigator assessment at Week 4, the median duration of effect (ie, the time to return to baseline glabellar line score at maximum frown by investigator assessment) was estimated to be 24.1 weeks for the 40-U dose, and the duration for the 20-U dose was 19.7 weeks. In this study, a 22.3% longer median duration of effect was achieved when the dose of onabotulinumtoxinA was doubled to 40 U from the approved dose of 20 U. The second was also a phase 2 dose-ranging study, in this case investigating single doses of placebo and 20 U, 40 U, and 60 U daxibotulinumtoxinA-lanm.^14^ In this study, the median duration of effect, based on a ≥ 1-point improvement from baseline in the glabellar line score at maximum frown by investigator assessment, was estimated to be 23.6 weeks for the 40-U dose; in comparison, the duration for the 20-U dose was 20.0 weeks. That is, an 18.0% longer median duration of effect was achieved when the dose of daxibotulinumtoxinA-lanm was doubled from 20 U to 40 U. Of note, a plateau seemed to have been reached in both of these dose-ranging studies in which no further benefit was observed with higher doses of either onabotulinumtoxinA or daxibotulinumtoxinA-lanm.^9,14^

Two subsequent placebo-controlled phase 3 40 U daxibotulinumtoxinA-lanm studies were also conducted.^18-20^ For these later studies, the median durations of effect for those achieving a ≥ 1-point improvement from baseline in their glabellar lines at maximum frown by investigator assessment were estimated to be 24.1 weeks for each of these studies.^18^ More recently, additional duration of effect data have been published for these studies, albeit not for a comparable endpoint.^19^ In this case, the median durations of effect, based on a return to their baseline glabellar line score of moderate or severe at maximum frown by both investigator and patient assessment, were estimated to be 27.7 and 26.0 weeks. This approach, requiring both investigator and patient to agree on the return to baseline severity, led to more prolonged estimates of duration than those reported earlier based on a ≥ 1-point improvement by investigator alone.

No studies have been published examining 40-U doses of either abobotulinumtoxinA or incobotulinumtoxinA; however, duration of effect data does exist for higher than approved doses of each. As above, the median duration of effect of 26.1 weeks reported for 2× the approved dose of prabotulinumtoxinA-xvfs also appears to compare favorably with durations reported in studies describing investigation of these other toxins at doses of 2 to 2.5× the approved doses, that is, at doses that most closely match a doubling of the approved dose.^10-12^ The first was an open label study describing investigation of a single 120-U dose of abobotulinumtoxinAm, that is, at 2.4× the approved dose of 50 U.^10^ The median duration of effect was 150 days (24.1 weeks), based on a return to a glabellar line score of moderate or severe at maximum frown by investigator assessment. While a phase 2 dose-ranging study reporting on investigation of single doses of placebo and 50 U, 75 U, 100 U, and 125 U abobotulinumtoxinA was also conducted, the reported duration of effect data were not directly comparable to our data set.^11^ Estimates of the duration of effect were not only based on concomitant investigator and participant agreement, but each utilized a unique assessment scale described respectively as an investigator live assessment photographic scale and a participant self-assessment static categorical scale. The last study of interest was a phase 2 dose-ranging study describing investigation of single doses of 20 U, 50 U, 75 U and 100 U incobotulinumtoxinA.^12^ In this study, the median duration of effect was estimated to be 185 days (26.4 weeks) for the 50 U dose, that is, 2.5× the approved dose.

These estimates of the median duration of effect (ie, the point in time at which 50% of patients have returned to their baseline scores and are no longer responders), as well as responder rates by various definitions at an arbitrary point in time, can serve as helpful tools when comparing outcomes across studies. Still, it is the responder rate over time data and graphs (Figures 6-12) that provides us the fuller picture of the investigator and patient assessments of the severity of the glabellar lines, their overall impression of the aesthetic benefit, and the patient's continued satisfaction with their treatment. While often not statistically significant, and regardless of the responder definition applied, differences in responder rates between 40 U prabotulinumtoxinA-xvfs and the 20 U controls were visually evident even out to Day 240 (34.3 weeks) and Day 270 (38.6 weeks), at which points either no or almost no patients in the control groups met the definitions of a responder.

When interpreting responder rate graphs, the achievement of a glabellar line severity score of either none or mild at maximum frown is a measure of considerable relevance because it is an outcome more reflective of clinical practice. That is, patients who continue to have no wrinkles or only mild wrinkles are usually not treated. It is only when that assessment progresses to moderate or severe that a patient typically returns to the clinic for further treatment. In the current study, the majority of patients who received the 40-U dose had severe glabellar lines at maximum frown at baseline: 70.6% by investigator assessment; 78.4% by patient assessment. It is inherently more difficult for more severe patients to become a responder when the definition is based on a score of none or mild at maximum frown. That is, severe patients must show a ≥ 2-point improvement in their glabellar lines to meet this responder definition, while moderate patients only need to exhibit a minimum 1-point improvement (from moderate to mild) for this same purpose. It is important to understand the baseline demographics within the cohorts of a study, or when comparing across studies, with a glabellar line severity score of none or mild as a responder definition.

One limitation of the current study was that no clinic visits were scheduled between Day 2 and Day 30. This was an intentional choice, with the intent of capturing Day 2 onset data but otherwise aiming to improve patient compliance over the yearlong study by limiting the visit schedule to 30-day intervals. It is acknowledged that this choice precludes any comparisons between treatments in the current study, or across studies, of response times in the early posttreatment phase (eg, at Days 7, 14, 21), a window of time that might otherwise have been of interest, particularly to patients who might be seeking an optimal response in the immediate posttreatment phase. It is further acknowledged that this study was a phase 2 pilot study and so was not powered for formal hypothesis testing; nonetheless, it did prove adequate for identifying a number of statistically significant differences between 40 U of prabotulinumtoxinA-xvfs and the 20 U controls. Still, further study of the 40-U dose in a larger patient population would be of benefit to confirm the results of the current study. Typical of glabellar line studies conducted for registration purposes, with 94.2% of study participants being female and 93.5% White, males and people with skin color were underrepresented in the current study. Without further study, the safety and duration of effect outcomes observed in the current study may not necessarily apply equally to these types of understudied populations.

## CONCLUSIONS

In this prospectively designed, multicenter, randomized, double-blind, active-controlled, increasing dose phase 2 pilot study, a doubled-dose 40-U hyperconcentrated formulation of prabotulinumtoxinA-xvfs, administered as a single treatment of 5 injections, 0.05 mL (8 U) per injection, total of 0.25 mL (40 U), was safe and effective for the treatment of moderate to severe glabellar lines in adult participants, with a median duration of effect of 6 months.
